# Successful intrahepatic cholangiocarcinoma conversion surgery after administration of fibroblast growth factor receptor inhibitor

**DOI:** 10.1007/s12328-024-02014-w

**Published:** 2024-07-10

**Authors:** Junichi Kaneko, Ryota Kiuchi, Masaki Takinami, Ippei Ohnishi, Jun Ito, Osamu Jindo, Masafumi Nishino, Yurimi Takahashi, Takanori Yamada, Takanori Sakaguchi

**Affiliations:** 1 Department of Gastroenterology, Iwata City Hospital, 512-3 Ookubo, Iwata, Shizuoka Japan; 2 Department of Gastroenterological Surgery, Iwata City Hospital, Iwata, Shizuoka Japan; 3 Department of Cancer Genome Medical Center, Iwata City Hospital, Iwata, Shizuoka Japan; 4 Division of Pathology, Iwata City Hospital, Iwata, Shizuoka Japan; 5 Department of Internal Medicine II, Hamamatsu University School of Medicine, Hamamatsu, Shizuoka Japan; 6 Department of Hepatology, Iwata City Hospital, Iwata, Shizuoka Japan

**Keywords:** Intrahepatic cholangiocarcinoma, Biliary tract neoplasm, Conversion surgery, Pemigatinib, Comprehensive genome profile

## Abstract

We describe a case of a 47-year-old male patient with initially unresectable intrahepatic cholangiocarcinoma of the right liver lobe with tumor thrombi extending from the right bile duct to the common and left bile ducts. Conventional chemotherapy with gemcitabine and cisplatin for 19 months resulted in progressive disease. Subsequently, a comprehensive genome profile revealed fibroblast growth factor receptor 2 rearrangement, and hence, pemigatinib administration was initiated. After 6 months of pemigatinib therapy, significant shrinking of the tumor and disappearance of the tumor thrombi in the common and left bile duct were observed. Subsequently, the patient underwent conversion surgery, resulting in successful radical resection of the tumor. The patient has been disease-free for 7 months.

## Introduction

Intrahepatic cholangiocarcinoma (iCCA) is an aggressive malignancy originating in the bile duct epithelium of the liver. Radical surgical resection is the only option for the long-term survival of patients with iCCA [1, 2]. Since iCCA progresses rapidly and is asymptomatic in the early stages, most patients with iCCA lose the opportunity for surgical treatment at the time of diagnosis [3]; therefore, only a limited number of patients can undergo surgery. Conventional chemotherapy regimens for iCCA have been developed that treat biliary tract cancers (BTC) as a whole [4]. Although improvements have been made in chemotherapy for unresectable BTC, its therapeutic effect remains insufficient.

Pemigatinib, a fibroblast growth factor receptor (FGFR) 1-, 2-, and 3-selective inhibitor, has been approved for the treatment of previously treated unresectable BTC with FGFR2 fusion or rearrangement [5–7], which is detected in 10–20% of iCCA patients [7]. Currently, case reports of patients treated with pemigatinib are still limited, and there are no reports of patients undergoing conversion surgery following pemigatinib administration [8, 9]. Herein, we report a case of initially unresectable iCCA that involved radical surgical resection following pemigatinib administration.

## Case report

**Fig. 1:**
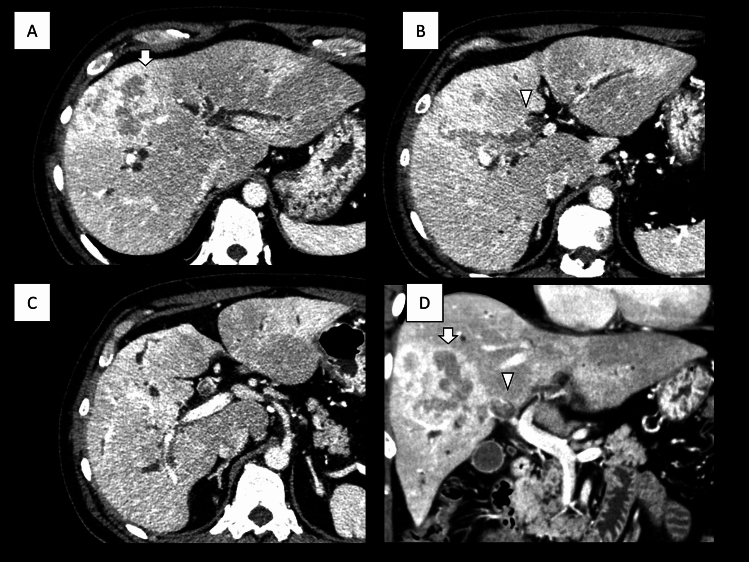
Abdominal computed tomography showing a 10 cm tumor in the right liver (white arrow), tumor invasion into the hilar bile duct, tumor thrombi in the common bile duct (white arrowhead), and dilated right and left intrahepatic bile ducts; **A**, **B**, and **C** are axial cross-sections; **D** is the coronal cross-section

**Fig. 2 A:**
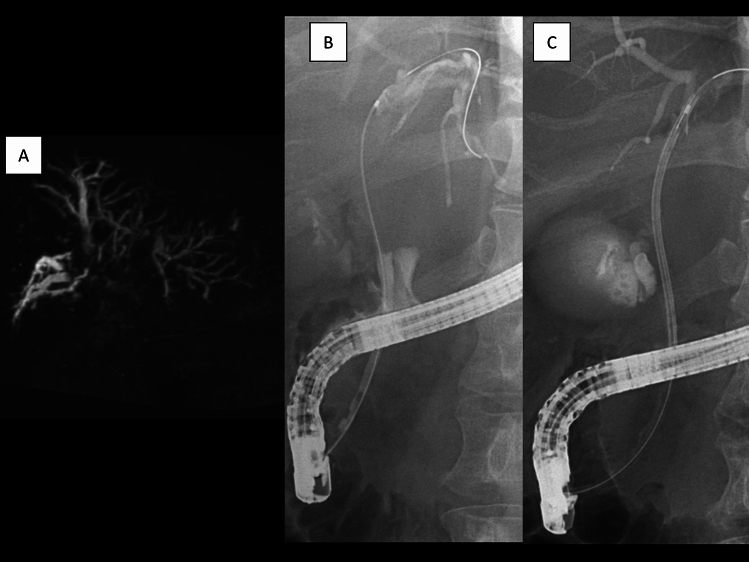
Magnetic resonance cholangiopancreatography showing a tumor obstructing both the right and left hepatic ducts; **B** fluoroscopy during endoscopic retrograde cholangiopancreatography showing tumor thrombus between the common bile duct and the left hepatic duct; **C** fluoroscopy during endoscopic retrograde cholangiopancreatography showing a plastic stent deployed to bridge the left and common bile ducts

A previously healthy 47-year-old male was referred to our hospital with complaints of epigastric pain and jaundice. Peripheral blood analysis revealed a white blood cell count of 9700 cells/μL, a total bilirubin level of 17.1 mg/dL, an aspartate aminotransferase level of 122 IU/L, an alanine aminotransferase level of 134 IU/L, and a C-reactive protein level of 11.81 mg/dL, indicating that the patient had no history of hepatitis B or C infection. Computed tomography (CT) showed a 10 cm tumor on the right side of the liver, tumor invasion into the hilar bile duct, tumor thrombi in the common bile duct, dilated right and left intrahepatic bile ducts, and tumor vascular invasion into the peripheral branches of the right portal vein, peripheral branches of the middle hepatic vein, and the right anterior hepatic artery (Fig. 1). Magnetic resonance cholangiopancreatography showed a tumor obstructing both the right and left hepatic ducts (Fig. 2A). Endoscopic retrograde cholangiopancreatography showed tumor thrombus between the common bile duct and the left hepatic duct. A plastic stent was placed to bridge the left and common bile ducts (Fig. 2B, C). Subsequently, peripheral blood tests were performed for tumor markers, revealing carbohydrate antigen 19-9 (CA19-9) at 242.4 U/mL (normal range < 37 U/mL), carcinoembryonic antigen at 3.2 ng/mL (normal range < 5 ng/mL), and alpha-fetoprotein at 2.4 ng/mL (normal range < 20 ng/mL). A percutaneous liver tumor biopsy was performed, and pathohistological analysis of the tumor revealed an adenocarcinoma arising from the biliary tract. Based on the above, the patient was diagnosed with locally advanced iCCA with vascular invasion and aggressive tumor thrombi. After discussions with multidisciplinary teams, the iCCA was deemed unresectable and chemotherapy was planned. In addition, the iCCA was classified as cT2N0M0 c-Stage II according to the 8th Union for International Cancer Control (UICC) TNM classification.

**Fig. 3:**
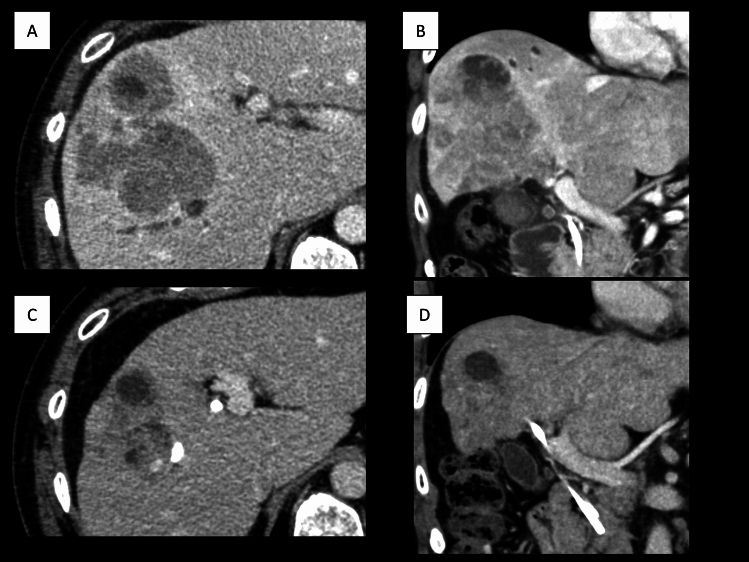
Abdominal computed tomography before pemigatinib treatment (**A**, **B**) and after 6 months of pemigatinib treatment (**C**, **D**); **A** and **B** show an increase in tumor size despite 19 months of conventional chemotherapy; **C** and **D** show tumor size reduction and the disappearance of tumor thrombi in the common bile duct after 6 months of pemigatinib treatment

**Fig. 4:**
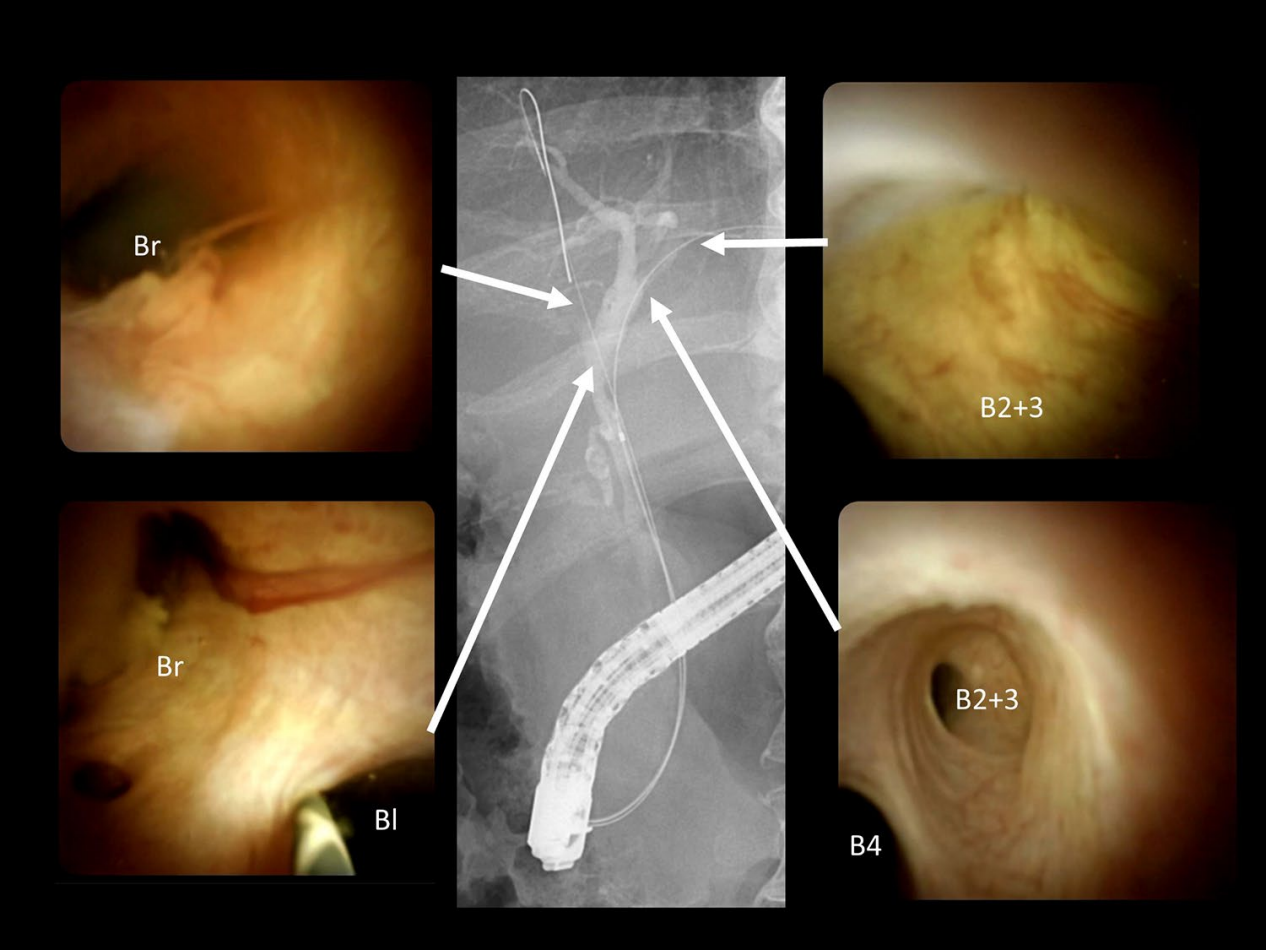
Peroral cholangioscopy showing no tumor invasion or visible tumor thrombi in the common bile duct, right hepatic duct (Br), left hepatic ducts (Bl) or left peripheral bile ducts (B4, B2 + 3) after 6 months of pemigatinib treatment

Gemcitabine (1000 mg/m^2^/day, days 1–8) and cisplatin (20 mg/m^2^/day, days 1–8) (GC) therapy was initiated and repeated every 3 weeks. Cisplatin was discontinued after 11 months of GC therapy owing to renal dysfunction, while gemcitabine (G) monotherapy continued. The effect of chemotherapy was evaluated by CT every 2–3 months and the tumor showed a gradual enlargement. Therefore, a comprehensive genome profile (CGP) was performed with patient consent to identify treatment options. FoundationOne CDx (Foundation Medicine) was submitted with liver biopsy specimens but failed due to insufficient specimen quality. Although it took 4 months after the initial CGP submission, the subsequent FoundationOne Liquid CDx (Foundation Medicine) revealed FGFR2 rearrangement. At that time, progressive disease was observed after 19 months of GC/G therapy due to an increase in tumor size to 12 cm according to the Response Evaluation Criteria in Solid Tumors (RECIST) version 1.1 (Fig. 3A, B). Therefore, pemigatinib (13.5 mg orally once a day for 14 consecutive days followed by 7 days off therapy, in 21-day cycles) was administered as a second-line treatment. Nail disorders and hyperphosphatemia were observed but were manageable with skincare and limited dietary phosphorus intake. After 6 months of pemigatinib therapy, a CT scan showed a significant reduction in the diameter of the largest liver lesion from 12 to 9 cm, and the tumor thrombi in the common and left bile duct had disappeared (Fig. 3C, D); in addition, CA19-9 decreased from 311.7 U/mL to 72.2 U/mL. Peroral cholangioscopy (POCS) showed no tumor invasion or visible tumor thrombi in the common bile duct, right hepatic duct, left hepatic ducts or left peripheral bile duct, and a POCS-guided targeted biopsy was negative for cancer (Fig. 4). The imaging studies showed no factors that would make the tumor unresectable, plus the tumor thrombi had shrunk and were confined to the right peripheral bile duct only. The treatment response was evaluated as a stable disease according to RECIST. After thorough discussions with multidisciplinary teams, the patient decided to undergo conversion surgery. A right hepatectomy, extrahepatic bile duct resection, choledochojejunostomy, and regional lymph node dissection were performed. Consequently, the patient underwent radical surgical resection, including frozen section evaluation. The patient was discharged on the 16th postoperative day with no postoperative complications.

**Fig. 5:**
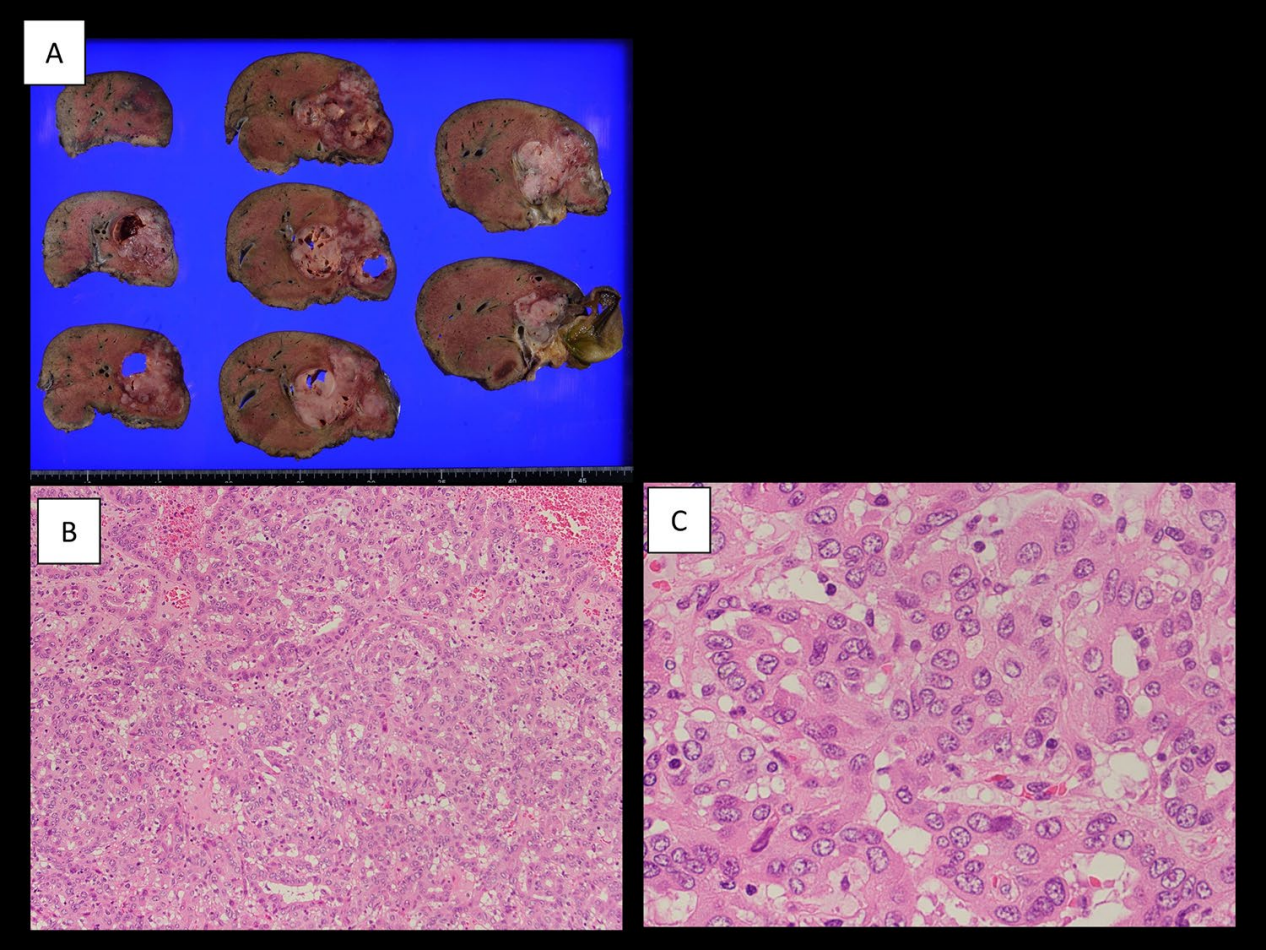
Macroscopical findings of the surgical specimen showing a grayish-white 9 × 8 cm tumor filling the lumen of the right bile duct and invaded the adjacent areas (**A**). Microscopical findings showing the tumor comprised atypical cuboidal cells in an irregular tubular pattern. **B** Hematoxylin and eosin staining × 10; **C** hematoxylin and eosin staining × 40

**Fig. 6:**
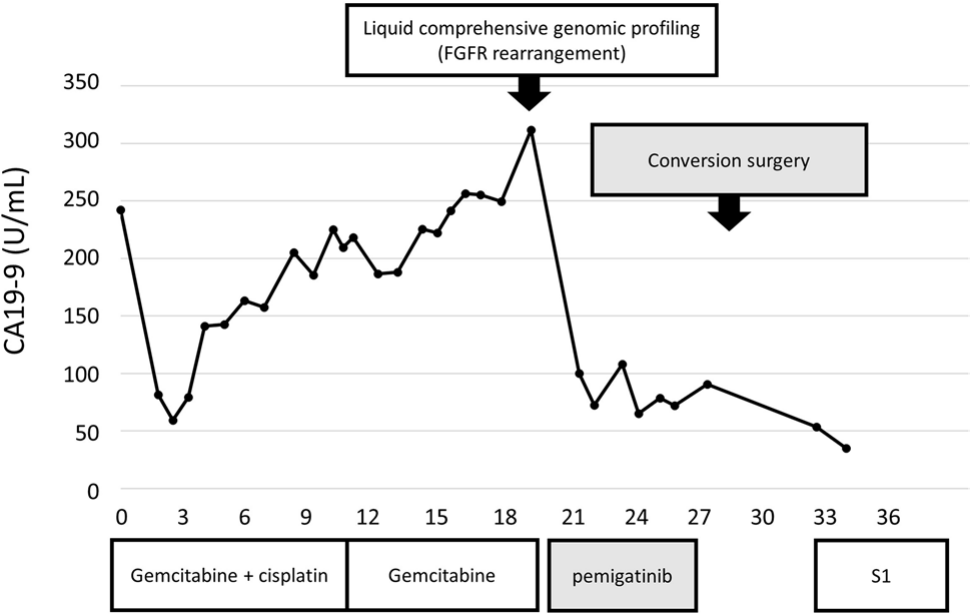
Trend of carbohydrate antigen 19–9 levels

Macroscopically, a 9 × 8 cm grayish-white tumor occupied the lumen of the right bile duct and invaded the adjacent areas (Fig. 5A). Histologically, the tumor comprised atypical cuboidal cells in an irregular tubular pattern (Fig. 5B, C). From these findings, the pathological diagnosis was well-differentiated adenocarcinoma as a subtype of iCCA. Tumor invasion into the peripheral portal vein (Vp1) was noted, but invasion into the hepatic vein was not observed. No serosal invasion was observed. Eight lymph nodes were dissected, and adenocarcinoma metastasis was found in one (12p LN in the hepatoduodenal ligament behind the portal vein). From these findings, the final tumor classification was pT2N1M0, p-Stage IIIB, according to the 8th UICC TNM classification. The pathological treatment effect was considered Grade I based on the Evans grading system [10]. The microscopic surgical margin was negative at the cutting surface of the liver and bile duct. As a postoperative adjuvant chemotherapy, oral S1 administration was performed for 6 months, and the patient had no recurrence 7 months after surgery. The trends in CA19-9 levels are shown in Fig. 6.

## Discussion

In this case report, we described a patient with initially unresectable iCCA who underwent successful radical surgical resection after pemigatinib administration.

Surgical resection is the only curative treatment for iCCA. A good indication for hepatic resection for iCCA is in cases with a solitary tumor in the periphery and a high-quality residual liver in the future [11]. However, there are no standard consensuses for the surgical indication of locally advanced BTC [12]. The Liver Cancer Study Group of Japan Clinical Practice Guidelines for Intrahepatic Cholangiocarcinoma indicate that vascular invasion or bile duct invasion more advanced than the right or left hepatic duct is associated with poor prognosis [4]. Particularly, surgical resection of iCCA involving the second-order biliary ducts bilaterally often requires extended hepatectomy, intrahepatic bile duct reconstruction, lymphadenectomy, and vascular resections that, in addition to the innate technical challenges, are associated with a high risk of postoperative complications and mortality [13, 14]. In the present case, a 10 cm tumor was mainly located in the right liver lobe, with no metastases, but with vascular invasion and aggressive tumor thrombi extending from the right bile duct to the common and left bile ducts. Therefore, the patient was diagnosed with locally advanced intrahepatic carcinoma with several poor prognostic factors and was comprehensively diagnosed as unresectable.

Bismuth et al. initially reported conversion surgery for hepatopancreatic-biliary malignancies in 1996 [15]. Recently, several cases have been reported in which initially unresectable BTCs were resected during conversion surgery [16–27]. Noji et al. conducted a multicenter retrospective study in Hokkaido, Japan, finding that the 1-, 3-, and 5-year survival rates following conversion surgery were 75.0%, 48.1%, and 38.2%, respectively, and that the 5-year survival rate of the 24 patients who received both chemotherapy and surgery was significantly better than that of 110 patients treated with chemotherapy only (*p* < 0.001) [27]. Since surgical resection is the only potentially curative treatment for iCCA, conversion surgery after chemotherapy would be a reasonable treatment to potentially improve patient outcomes; however, published reports on conversion therapy for iCCA are limited, and the heterogeneity of therapeutic agents currently limits comparative evaluations.

GC therapy has been the standard first-line chemotherapy for advanced BTC since the publication of the ABC-02 study [28]. However, the median overall survival with GC therapy is 11.7 months, while the 24-month survival rate is approximately 15%, suggesting the need for new therapies. Recently, gemcitabine and S-1 plus cisplatin (GCS) therapy was demonstrated to have survival benefits and a higher response rate than GC therapy in a randomized phase III trial (KHBO1401-MITSUBA) [29]. In a double-blind, placebo-controlled, phase 3 study to evaluate chemotherapy in patients with advanced BTC (TOPAZ-1 trial), durvalumab plus chemotherapy significantly improved overall survival and showed improvements compared to placebo plus chemotherapy [30]. These studies indicate that primary chemotherapy for BTC is shifting from the GC era to an era of new therapeutic agents.

Pemigatinib, a selective and potent oral inhibitor of FGFR1, 2, and 3, offers promise for treating unresectable BTC. An open-label, multicenter, phase II study (FIGHT-202) showed that among patients with FGFR2 fusions or rearrangements who were treated with pemigatinib following first-line therapy, 35.5% (38/107) achieved an objective response (three complete responses and 35 partial responses); median time to first response was 2.7 months (IQR 1.4–3.9); duration of response (DOR) among responders was 7.5 months (95% CI 5.7–14.5) [5]. These results support the approval of pemigatinib in the United States, Japan, and Europe for patients with previously treated unresectable locally advanced or metastatic BTC with FGFR2 fusion or rearrangements. FGFR2 fusions or rearrangements have also been reported to occur almost exclusively in patients with iCCA, making them a particularly promising target for iCCA treatment; however, to the best of our knowledge, this is the first reported case of conversion surgery after pemigatinib administration.

Futibatinib, a next-generation, covalently binding FGFR1-4 inhibitor, was recently shown to have antitumor activity in patients with FGFR2 fusion or rearrangement-positive tumors and was adopted in Japan. A phase II, open-label, multicenter study of futibatinib in patients with iCCA harboring FGFR2 gene fusions or other rearrangements (FOENIX-CCA2) showed that a total of 43 of 103 patients (42%) had a response, and the median DOR was 9.7 months (95% CI 7.6–17.0) [31]. Therefore, futibatinib is a promising treatment for patients with FGFR2 fusion or rearrangement-positive iCCA. Further research is needed to determine how these FGFR inhibitors should be used differently.

Recently, CGP has been rapidly incorporated into the clinical management of cancer. BTC is enriched in a relatively high number of actionable mutations, including those in FGFR. A previous study showed that the percentages of Asian and Western patients who had ≥ 1 actionable genetic aberration (GA) were 72.0% and 60.9%, respectively [32]. This indicates that CGP is a promising test for the treatment of unresectable BTC. Currently, patients with solid tumors who have completed (or are expected to complete) standard treatment are eligible for CGP. Early CGP may help achieve optimal treatment earlier. In addition, CGP has a long turnaround time; both FoundationOne CDx and FoundationOne Liquid CDx typically require 10 days or more to provide results. When FoundationOne CDx Liquid is used after a failed FoundationOne CDx, as in our case, it may take > 1 month to obtain results. Hence, the optimal time for submitting a CGP may be an issue to be focused on in future studies. In the present case, conversion surgery was performed 6 months after pemigatinib administration. Given the median DOR of 7.5 months among responders in the FIGFT202 trial [5], performing conversion surgery 6 months after pemigatinib administration may be reasonable; however, the optimal timing of conversion surgery after pemigatinib administration remains unclear. To resolve these clinical questions, it is essential to gather cases where patients underwent surgery after pemigatinib administration.

In conclusion, we report the case of a patient with initially unresectable iCCA who underwent successful radical surgical resection after pemigatinib administration. CGP can be a game-changer for iCCA treatment; however, many issues need to be resolved, including the optimal timing of CGP submission and conversion surgery.
